# Tumor Contrast-Enhancement for Monitoring of PRRT ^177^Lu-DOTATATE in Pancreatic Neuroendocrine Tumor Patients

**DOI:** 10.3389/fonc.2020.00193

**Published:** 2020-02-21

**Authors:** Olof Pettersson, Katarzyna Fröss-Baron, Joakim Crona, Anders Sundin

**Affiliations:** ^1^Section of Radiology, Department of Surgical Sciences, Uppsala University, Uppsala, Sweden; ^2^Department of Medical Sciences, Uppsala University, Uppsala, Sweden

**Keywords:** therapy monitoring, neuroendocrine tumor, computed tomography, contrast-enhancement, NET, CT, PRRT, ^177^Lu

## Abstract

**Background:** Therapy monitoring of cancer treatment by contrast-enhanced CT (CECT), applying response evaluation criteria in solid tumors criteria version 1. 1 (RECIST 1.1) is less suitable for neuroendocrine tumors (NETs) which, when responding, tend to show stabilization rather than shrinkage. New methods are needed to further classify patients in order to identify non-responders at an early stage and avoid unnecessary adverse effects and costs. Changes in arterial tumor attenuation and contrast-enhancement could be used to identify the effect of therapy, perhaps even in early stages of treatment.

**Methods:** Patients with metastatic pancreatic NETs (PNETs) receiving peptide receptor radionuclide therapy (PRRT) with ^177^Lu-DOTATATE underwent CECT at baseline, mid-treatment (PRRT cycles 3–5) and at follow-up, 3 months after the last PRRT cycle. At baseline CECT, the liver metastasis with the highest arterial attenuation was identified in each patient. The fold changes in arterial tumor attenuation (Hounsfield Units, HU), contrast-enhancement (HU), and transversal tumor area (cm^2^) between CECT at baseline, mid-treatment and follow-up were calculated. Correlation of the tumor metrics to outcome parameters such as progression-free survival (PFS) and time to best response was performed.

**Results:** Fifty-two patients were included (27 men, 25 women), median age 60 years (range 29–80), median Ki-67 8% (range 1–30). Six patients had grade 1 PNETs, forty had grade 2 and four had grade 3 tumors. As an internal control, it was first tested and established that the tumor contrast-enhancement was not merely related to that of the abdominal aorta. The mean ± SD arterial attenuation of the liver metastases was similar at baseline, 217 ± 62 HU and at mid-treatment, 238 ± 80 HU and then decreased to 198 ± 62 HU at follow-up, compared to baseline (*p* = 0.024, *n* = 52) and mid-treatment (*p* = 0.0004, *n* = 43). The transversal tumor area decreased 25% between baseline and follow-up (*p* = 0.013, *n* = 52). Tumor contrast-enhancement increased slightly from baseline to mid-treatment and these fold changes correlated with PFS (*R*^2^ = 0.33, *p* = 0.0002, *n* = 37) and with time to best response (*R*^2^ = 0.34, *p* < 0.0001, *n* = 37).

**Conclusions:** Early changes in contrast-enhancement and arterial attenuation in PNET liver metastases may for CECT monitoring of PRRT yield complementary information to evaluation by RECIST 1.1.

## Introduction

The pancreatic neuroendocrine tumor (PNET) constitutes a subgroup of the gastroenteropancreatic neuroendocrine tumors (GEP-NETs) which derive from local multipotent gastrointestinal stem cells of the diffuse neuroendocrine cell system (DNES). They are relatively rare and due to a lack of consensus on their classification, the epidemiology has been difficult to ascertain. In a European population, the incidence for a wide spectrum of GEP-NETs ranges from 1 to 5 per 100,000 persons per year (1–5). Similar incidences where found in the US, while the specific incidence for pancreatic NETs in the same population was <1 per 100,000 persons per year (6).

PNETs are divided into functional tumors, giving rise to a hormonal syndrome, and non-functioning tumors which do not, although hormonal activity can be detected biochemically. Among the functioning PNETs, insulinomas are the most frequent and are often benign, whereas gastrinomas and glucagonomas, frequently metastasize to the liver, lymph nodes and bone. In general, PNETs are large at diagnosis and approximately half of them have metastasized. Patients with functional PNETs are, however, usually detected at an earlier stage because of hormonal symptoms. Non-functioning tumors may cause some abdominal discomfort, but typically give little or no symptoms (1, 7–9).

Contrast-enhanced computed tomography (CECT) is the most readily available modality for the primary diagnostic workup and staging of PNETs, for surveillance and to monitor therapy. Magnetic resonance imaging (MRI) is recognized as superior to CT for evaluation of the liver, bile ducts, and pancreas, and allows for diffusion-weighted imaging (DWI) and the use of hepatocyte-specific contrast media. Nevertheless, CT is generally sufficiently effective for imaging of PNETs and as the availability of CT is much higher than that of MRI, even at academic institutions, CT is the golden standard for therapy monitoring (2, 9–12).

Monitoring of oncological therapies in the vast majority of patients relies on assessing changes of tumor sizes on CT/MRI by applying the Response Evaluation Criteria in Solid Tumors version 1.1 (RECIST 1.1) (13, 14). For functional therapy monitoring, PET/CT with ^68^Ga-DOTA-somatostatin analogs (^68^Ga-DOTA-SSAs) have in conjunction with PRRT of GEP-NETs been shown to detect new lesions earlier than CECT, but changes in tumor ^68^Ga-DOTA-SSA uptakes over time have not yet convincingly been shown to reflect therapy outcome (3, 11).

In cases of locally advanced inoperable or metastasized PNETs, systemic treatment is generally initiated. G1 and low G2 PNETs (Ki-67 index ≤ 5%) may be treated with somatostatin analogs and tumors of higher grade with chemotherapy or targeted molecular agents, such as mTOR inhibitors and tyrosine kinase inhibitors. During recent years, PRRT with ^177^Lu-DOTATATE is increasingly used when other therapies have failed. Treatment may be repeated after a few years in case of disease progression, so called “salvage therapy” administering a reduced number of cycles (15–19).

Response to GEP-NET therapies rarely results in marked tumor shrinkage, but rather stabilization, and CT/MRI-monitoring of treatment response with the RECIST 1.1 criteria is therefore troublesome (3, 10, 14, 20). Even the effects of PRRT with ^177^Lu-DOTATATE on tumor size are at best discreet and generally appear late, with best response often seen after about a year. There is therefore a need to develop new methods to assess therapy outcome, if possible at an early stage, in order to stop inefficient treatments and avoid undesirable side effects and costs. Tumor cell death and micro-necrosis, with related effects on tumor vascularity, are likely to occur much earlier than tumor shrinkage. Changes in the arterial tumor attenuation (Hounsfield Units, HU) and contrast-enhancement on CECT, reflecting tumor vascularity, may therefore be suitable early markers of therapy response (or lack thereof), in parallel to monitoring of tyrosin kinase inhibitor therapy of gastrointestinal stromal tumors (GIST), according to the Choi criteria (21).

In this report, we evaluated the fold changes of the arterial attenuation and the contrast-enhancement in hypervascular PNET liver metastases on CECT during and after ^177^Lu-DOTATATE treatment, in relation to baseline and with correlation to PRRT outcome parameters. We hypothesize that PNET liver metastases in response to PRRT also undergo vascular changes which can be visualized in the arterial phase of CECT, and that these changes may correlate to time to best response and progression-free survival (PFS).

## Materials and Methods

### Patients

The study was approved by the Local Ethics Committee for Human Ethics in Uppsala, Sweden (reference number: 2010/177) and all patients provided written informed consent. The declaration of Helsinki was followed. All patients with a PNET diagnosis who had undergone PRRT with ^177^Lu-DOTATATE at Uppsala University Hospital between 2006 and 2018 (*n* = 151) were identified in the hospital's radiological information system (RIS) and picture archiving and communication system (PACS). Only patients with at least one hypervascular liver metastasis on CECT were included. Those with partly calcified metastases or metastases with extended tumor necrosis were excluded, due to the risk of partial volume effect in the attenuation measurements. Also, patients lacking relevant CECT before and/or after treatment were excluded as were those in which the timing of the CT scanning in relation to contrast medium administration was inadequate (e.g., venous phase instead of late arterial phase). Furthermore, patients who had received insufficient amounts of contrast medium were excluded. In general, patients examined with a CECT of poor technical quality were excluded.

Baseline CECT was performed within 1 month before PRRT, which comprised 7,4 GBq per treatment cycle, and the number of cycles were tailored according to kidney and bone marrow dosimetry (15). During PRRT, CECT was performed before the fifth cycle (if available, otherwise before the third cycle), henceforth referred to as “mid-treatment.” Follow-up CECT was undertaken at preferably 3 months after the last cycle and at the latest 6 months, mean ± SD 3.0 ± 1.3 months after the last PRRT cycle. Patients with follow-up CECT later than 6 months after PRRT were excluded.

Data on Ki-67 and tumor grade were collected from the pathologists' reports on the biopsies of the liver metastases, if available. Data on chromogranin-A at baseline was collected from the laboratory reports.

### CECT Measurements

For each patient, the liver metastasis with the highest attenuation in the late arterial contrast-enhancement phase at baseline CECT (assessed by visual inspection) was outlined manually using an irregular region of interest (ROI) and its mean attenuation (HU), maximum attenuation (HU) and transversal surface area (cm^2^) was noted. This was also transferred to the corresponding non-contrast-enhanced images.

The fold changes (%) of the arterial tumor attenuation, contrast-enhancement and transversal tumor area on CECT at baseline to follow-up, between baseline and mid-treatment and between mid-treatment and follow-up were calculated. The combined fold changes in arterial tumor attenuation and transversal tumor area between these time points were also assessed.

A ROI was also placed in the abdominal aorta at the level of the coeliac trunk to achieve an approximate measurement of the attenuation in the hepatic arterial branches, from which the tumor vessels of the liver metastases are derived. Aortic contrast-enhancement was assessed at baseline and at follow-up, in order to exclude that the fold changes in arterial tumor attenuation and contrast-enhancement not merely reflected those in the aorta. Thus, it was established that the changes in lesion attenuation and contrast-enhancement reflected biological effects, such as changes in vascularity, as a response to PRRT, and not merely variations in aortal attenuation.

### Outcome Parameters

The CECT measurements were correlated with the outcome parameters best response (BR) according to RECIST 1.1, time to BR, progression-free survival (PFS) and overall survival (OS). OS was defined as the interval from initiation of therapy until death or the last day of follow-up.

The patients underwent clinical, biochemical and radiological assessment by CECT at least every 3–6 months following PRRT. CECT from baseline was assessed according to RECIST 1.1 and the maximum percentage decrease/increase in the sum of tumor diameters (maximum two per organ, maximum five in total) was calculated (BR RECIST 1.1) as well as the time to BR. A twenty percent increase in the sum of tumor diameters and/or the appearance of new tumor lesions was categorized as progressive disease and, together with the patients' clinical and biochemical data, allowed for establishing PFS.

Only hypervascular liver metastases were assessed for fold changes in arterial attenuation, contrast-enhancement and size. The complete RECIST 1.1 measurements were used as a means to evaluate outcome.

### Statistical Methods

Statistical analysis was performed with JMP 14 (SAS Institute Inc., Cary, NC, USA) software. Wilcoxon's non-parametric test was used to analyze differences in the fold changes between baseline and follow-up in arterial attenuation and contrast-enhancement, respectively, in the liver metastases. Correlations between CECT data and BR RECIST 1.1, time to BR, PFS, and OS were tested by linear regression.

## Results

### Baseline Patient Characteristics

**Figure 1:**
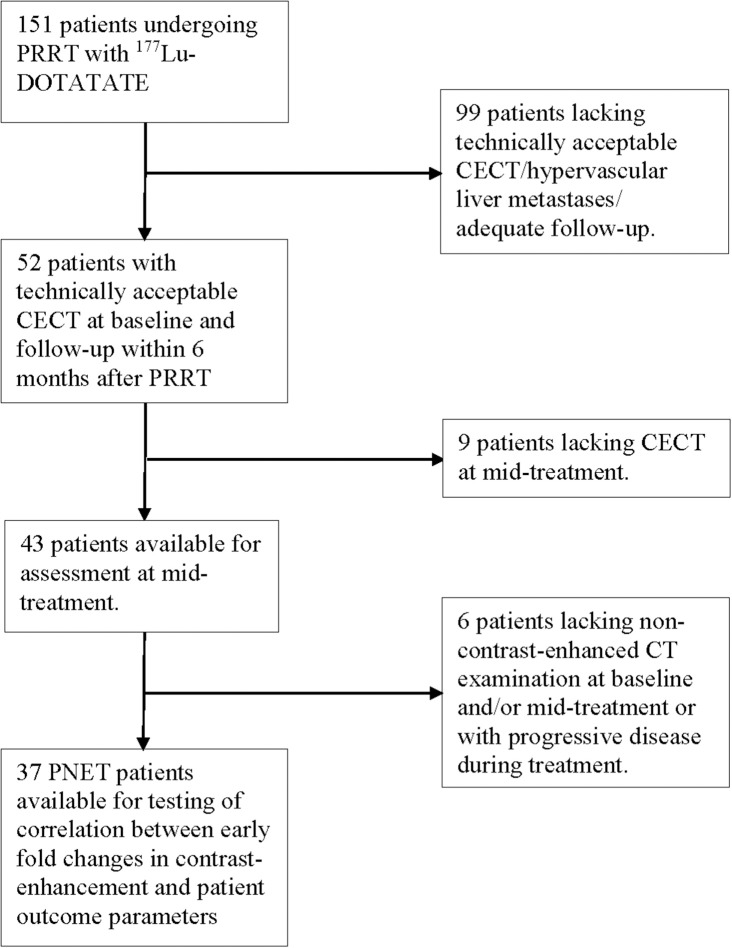
Flow chart depicting the number of PNET patients included for the different evaluations.

Out of the original 151 patients, 52 had liver metastases that were available for inclusion (Figure 1). Thus, the study cohort comprised 52 patients (27 men, 25 women) with median age 60 years (range 29–80) at the start of therapy. Ki-67 index was available in 50 patients, median at 8% (range 1–30). In the majority of patients (*n* = 45), Ki-67 was reported from biopsies of liver lesions, otherwise from the primary tumor (*n* = 5). Six patients had grade 1 PNETs, 41 had grade 2, and three subjects harbored grade 3 tumors. As the vast majority of patients harbored grade 2 tumors, and only a few had grade 1 or grade 3 tumors, no subsequent subgroup analysis was performed.

**Figure 2:**
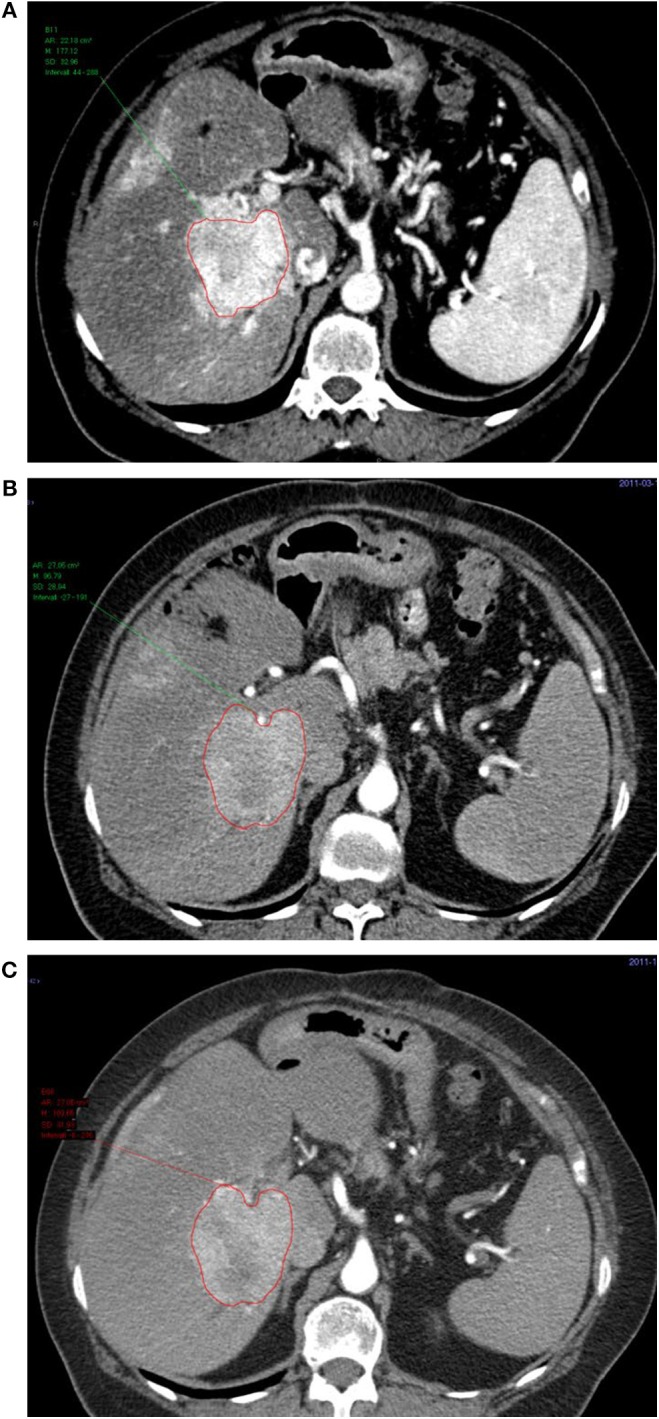
Transversal contrast-enhanced CT images in the arterial phase of a liver metastasis at baseline **(A)**, before the 3rd cycle of treatment **(B)**, and at follow-up **(C)**.

The patients harbored at least one hypervascular liver lesion (Figure 2) and except for liver metastases there were metastases to lymph nodes (*n* = 20), bone (*n* = 9), adrenal glands (*n* = 3), ovaries (*n* = 1), peritoneum (*n* = 1), and bile ducts (*n* = 1). Direct extension of the primary PNET with tumor infiltration of the stomach (*n* = 2) and the spleen (*n* = 2) was also found.

Chromogranin-A was available in 48 patients, median 21 nmol/L (range 2 nmol/L to a 168-fold on the upper reference limit). All patients showed high tumor uptake on somatostatin receptor imaging (Krenning score 3–4).

### PRRT With ^177^Lu-DOTATATE

The majority of patients (*n* = 32) underwent three to five cycles of therapy. Nine patients received six cycles of therapy, whereas three went through seven cycles and a single patient was given 10 cycles. Among the remaining seven patients, the majority (*n* = 6) underwent two cycles of therapy. One patient underwent received only a single cycle of PRRT.

### Aortic Attenuation and Enhancement

**Figure 3:**
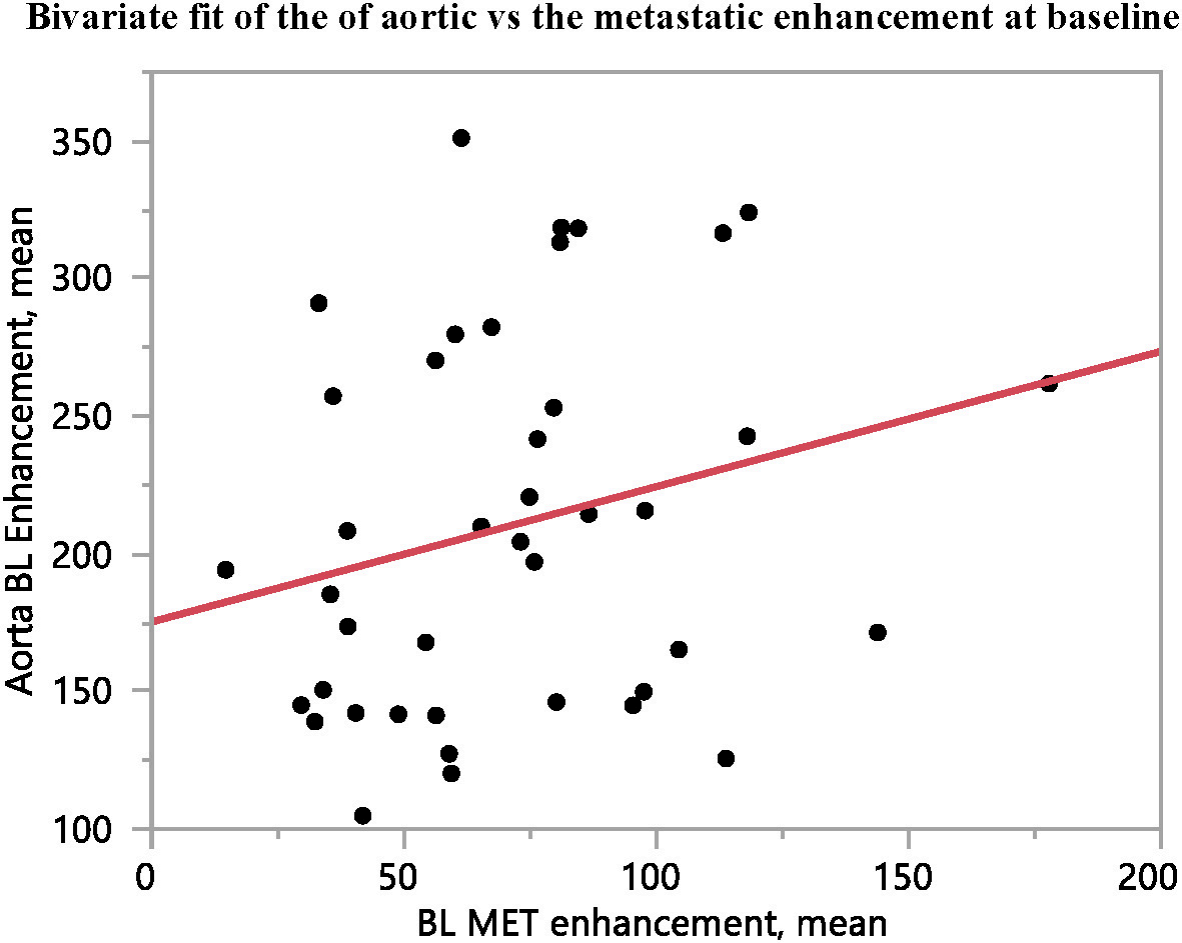
The contrast-enhancement in the abdominal aorta at the level of the coeliac trunk shows no correlation to the contrast-enhancement of the metastases at baseline (*n* = 41, *R*^2^ = 0.06, *p* = 0.12).

**Figure 4:**
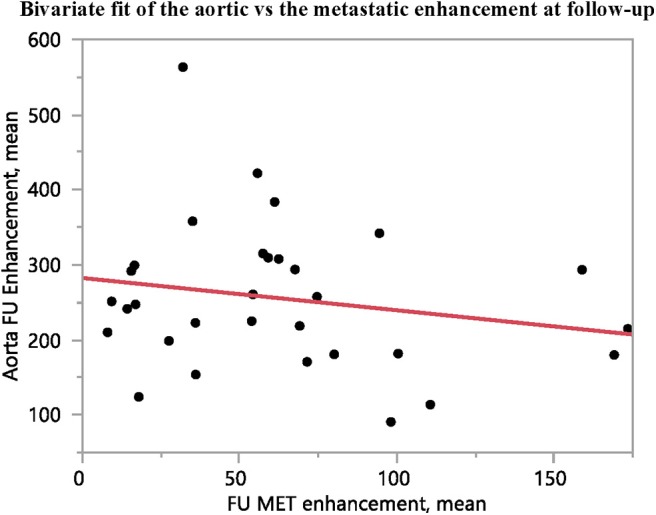
The contrast-enhancement in the abdominal aorta at the level of the coeliac trunk shows no correlation to the contrast-enhancement of the metastases at follow-up (*n* = 31, *R*^2^ = 0.04, *p* = 0.28).

As an internal control for tumor contrast-enhancement, aortic contrast enhancement at the level of the coeliac trunk was assessed, to verify that the tumor contrast-enhancement was not merely a reflection of that in the aorta. There was no correlation between the contrast-enhancement in the metastases and that in the aorta, at either baseline or follow-up (Figures 3, 4).

### Arterial Tumor Attenuation and Tumor Area

Out of 52 patients for whom fold changes in arterial attenuation and transversal tumor area between baseline and follow-up was measured, 43 subjects were available for measurements also of arterial attenuation at mid-treatment before the 3rd (*n* = 23) or before the 5th therapy cycle (*n* = 20) (Figure 1).

The maximum arterial attenuation in the metastases at baseline, 217 ± 62 HU, decreased significantly at follow-up, 198 ± 62 HU (*p* = 0.025, *n* = 52) and also from mid-treatment, 238 ± 80 HU, to follow-up 197 ± 64 HU (*p* = 0.0004, *n* = 43), but not between baseline and mid-treatment (*p* > 0.05, *n* = 43). Interestingly, there was a slight increase in arterial tumor attenuation from baseline to mid-treatment (*p* > 0.05) (Table 1).

**Table 1:** Tumor area, attenuation, and contrast-enhancement at baseline, mid-treatment, and follow-up.

**Tumor parameter**	**Baseline (mean ± SD)**	**Mid-treatment (mean ± SD)**	**Follow-up (mean ± SD)**	**Number of analyzed patients (*n*)**
Transversal area	16 ± 17 cm^2^	12 ± 12 cm^2^	12 ± 15 cm^2^	52
Maximum arterial attenuation	217 ± 62 HU	238 ± 80 HU	198 ± 62 HU	52 (43 at mid-treatment)
Maximum contrast-enhancement	107 ± 53 HU	114 ± 68 HU	101 ± 72 HU	37 (28 at follow-up)

*HU, Hounsfield Units*.

There was a significant decrease in the transversal tumor area in the arterial phase from baseline (mean ± SD 16 ± 17 cm^2^) to follow up (12 ± 15 cm^2^) (*p* = 0.013, *n* = 52).

### Correlation of CT-Parameters With Therapy Outcome

**Figure 5:**
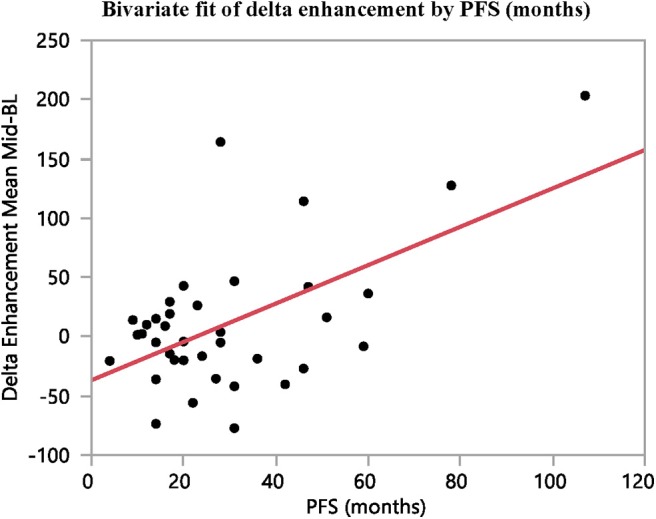
The difference in contrast-enhancement during early stages of treatment (baseline to mid-treatment) correlates with PFS (*n* = 37, *R*^2^ = 0.33, *p* = 0.0002).

**Figure 6:**
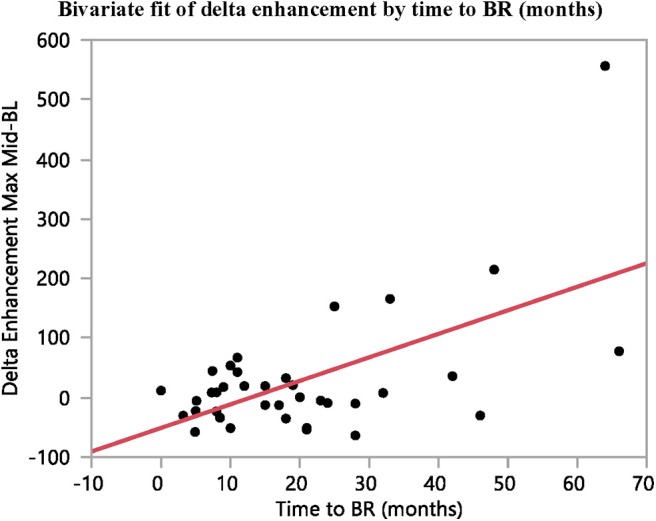
The difference in contrast-enhancement during early stages of treatment (baseline to mid-treatment) correlates with time to BR (*n* = 37, *R*^2^ = 0.34, *p* = 0.0001).

The results of the bivariate analyses are shown in Table 2 and Figures 5, 6. CT data vs. the therapy outcome parameters BR RECIST 1.1, time to BR and PFS. Testing of fold changes in transversal area and attenuation, respectively, vs. outcome parameters was possible in 45 patients, whereas contrast-enhancement data were available for 37 patients.

**Table 2:** Correlations between input data and outcome parameters.

**Tumor measurements**	**Outcome parameter**	**Interval**	***R*^**2**^**	***P***	**Number of analyzed patients (*n*)**
Transversal area	Best response	Baseline to follow-up	0.14	0.0028	45
Mean arterial attenuation	Best response	Baseline to follow-up	0.02	0.3335	45
Maximum arterial attenuation	Best response	Baseline to follow-up	0.05	0.1507	45
Maximum contrast-enhancement	Time to best response	Baseline to mid-treatment	0.34	<0.0001	37
Maximum contrast-enhancement	PFS	Baseline to mid-treatment	0.26	0.0014	37
Maximum contrast-enhancement	OS	Baseline to mid-treatment	0.13	0.0260	37
Mean contrast-enhancement	Time to best response	Baseline to mid-treatment	0.28	0.0008	37
Mean contrast-enhancement	PFS	Baseline to mid-treatment	0.33	0.0002	37
Mean contrast-enhancement	OS	Baseline to mid-treatment	0.16	0.0129	37
Mean arterial attenuation and area	PFS	Baseline to mid-treatment	0.11	0.0309	43
Mean arterial attenuation and area	Time to best response	Baseline to mid-treatment	0.09	0.0461	43

*PFS, progression-free survival; OS, overall survival*.

A strong correlation was found between PFS and time to BR (*R*^2^ = 0.85, *p* < 0.0001, *n* = 43) and between PFS and OS (*R*^2^ = 0.77, *p* < 0.0001, *n* = 46), whereas no correlation was found between BR RECIST% and PFS or BR RECIST% and time to BR (data not shown).

The therapy outcome parameters BR RECIST 1.1, time to BR and PFS were correlated to arterial attenuation, contrast-enhancement and tumor area. Regression analysis between BR and the fold changes in transversal tumor area from baseline to follow-up yielded no convincing correlation (*R*^2 =^ 0.14, *p* = 0.0028, *n* = 45). Similar results were found between the combination of fold changes in arterial attenuation and tumor area and PFS and time to BR, but no convincing correlations were found.

The arterial tumor attenuation and the contrast-enhancement were found to increase between baseline and mid-treatment, although not significantly. However, the fold changes in contrast-enhancement between these examination points were found to correlate with both PFS and time to best response (*R*^2^ = 0.33, *p* = 0.0002; and *R*^2^ = 0.34, *p* < 0.0001; *n* = 37, respectively). No reliable correlation could be found between the fold changes in contrast-enhancement and OS (*R*^2^ = 0.16, *p* = 0.0129, *n* = 37).

Overall, moderate correlations were found between the differences in contrast-enhancement from baseline to mid-treatment and time to BR (*R*^2^ = 0.34, *p* < 0.0001) and PFS (*R*^2^ = 0.33, *p* = 0.0002) (Table 2 and Figures 5, 6).

## Discussion

In cases of locally advanced inoperable PNET or disseminated disease, systemic treatment is often initiated. During recent years, PRRT with ^177^Lu-DOTATATE is increasingly used when other regimens, such as chemotherapies or targeted molecular agents, have failed (15–19).

As this is both very costly and carries some adverse effects, it is of great importance that the means for therapy monitoring are reliable and, if possible, allow for identification of non-responders at an early stage. Therapy monitoring of NETs traditionally relies on changes in tumor size on CT/MRI according to RECIST 1.1 (2, 3, 7, 10, 22). However, NETs tend to stabilize rather than shrink, in response to therapy, and favorable therapy response may be difficult to detect and thus become apparent only after several months of therapy. Conversely, therapy failure with early progression of NETs can escape detection when therapy monitoring only relies on RECIST 1.1 criteria. Moreover, many aspects of tumor biology are not included in the RECIST criteria, such as tumors undergoing cystic changes and necrosis as a consequence of therapy (2, 3, 10, 14, 20). This has previously been studied in treatment of GIST with imatinib and has for this tumor type lead to development of adapted response criteria (20, 21, 23, 24).

In early retrospective evaluations of PRRT of neuroendocrine tumors, promising clinical benefits and low toxicity were reported, despite little reduction in tumor size (25). These findings have subsequently been confirmed, with similarly promising data, also in patients undergoing salvage therapy (26–29).

^68^Ga-DOTATOC PET has shown valuable to detect new tumors during PRRT and may have a role in predicting PFS as indicated by assessment of fold changes of the tumor-to-spleen ratio (30, 31). Fold changes in ^68^Ga-DOTA-SSA tumor uptake during PRRT should, however, be interpreted with caution because the uptake may be influenced by several factors, other than the therapy itself, such as simultaneous SSA treatment and the amount of administered peptide in the ^68^Ga-DOTATOC preparation (32). Furthermore, tumor uptake measurements of ^68^Ga-DOTATOC is unreliable for the lesions with a high uptake (SUV > 25) (33).

In an attempt to refine therapy monitoring in PNET patients undergoing PRRT, the present CECT study assessed the fold changes in arterial tumor attenuation, contrast-enhancement and transversal area of hypervascular liver metastases in correlation with the therapy outcome parameters BR RECIST 1.1, time to BR and PFS. As expected, the maximum arterial tumor attenuation decreased from baseline to follow-up (*p* = 0.024, *n* = 52). Interestingly, this effect was not distinguished as an early sign of response during ongoing therapy, but instead appeared later, from mid-treatment to follow-up, after PRRT (*p* = 0.0004, *n* = 43). This suggests that the major biological effects of PRRT were possible to assess only in the later stages of the therapy.

However, the changes in tumor contrast-enhancement seemed to occur earlier than that of lesion size, allowing for detecting therapy response earlier than by the RECIST 1.1 criteria. Interestingly, we found that the contrast-enhancement in the metastases increased during early stages of PRRT (baseline to mid-treatment), as opposed to the decreased arterial attenuation seen in the later stages of the therapy. This early increase of the contrast-enhancement was found to correlate moderately with PFS (*R*^2^ = 0.33, *p* = 0.0002, *n* = 43) and with the time to BR (*R*^2^ = 0.34, *p* < 0.0001; *n* = 37). One possible model for explaining the slight increase in contrast-enhancement in the liver metastases during early therapy could be parenchymal or vascular damage from the ^177^Lu beta radiation. The irradiated liver has previously been quite extensively studied and seems to mimic a form of veno-occlusive disease with hypoattenuating areas, but with none or little actual vessel occlusion (34–36). A rare example of post-radiation hypervascularity has been described in the context of stereotactic body radiotherapy of hepatocellular carcinoma, where it may be regarded as a pitfall in tumor response evaluation (37). Consequently, it appears unlikely that the observed increase in tumor contrast-enhancement during early therapy should be a consequence of irradiation. To the best of our knowledge, fold changes in tumor attenuation and contrast-enhancement has not previously been explored as a tool for PRRT monitoring.

The observed fold changes in contrast-enhancement were also tested for correlation with OS. This yielded no convincing results (*R*^2^ = 0.16, *p* = 0.0129, *n* = 37). While it may be possible to relate differences in contrast-enhancement to progression-free survival, it would be much more challenging to argue that these early changes in lesion vascularity could influence overall survival. Thus, one should be careful when interpreting any correlations between therapy monitoring and overall survival.

Treatment response was also evident from a decrease in the transversal tumor area from baseline to follow-up (*p* = 0.013, *n* = 52) but could not be used to monitor early responses.

A limitation of this retrospective study was that merely a third of our patients were available for inclusion. Although 151 PNET patients had undergone PRRT in our hospital, the inclusion criteria of CECT performed in a well-timed late arterial contrast-enhancement phase, at repeated time points, considerably reduced the number of patients available for assessment. Especially the number of patients for whom tumor contrast-enhancement could be evaluated, was limited, the main reason being that non-contrast-enhanced CT had not been performed. In the clinical routine of many radiology departments, non-contrast-enhanced studies of the liver are unfortunately not included in the CT protocol, despite the recommendations in previous and current guidelines (2, 9, 10). In order not to reduce the study group further, we chose to perform measurements in patients harboring at least one liver metastasis, although more information may have been gained by assessing several lesions. Moreover, it could be argued that tumor volume is a more representative measurement to monitor shrinkage, than the maximum diameter of the lesion. However, assessment of volume is more time-consuming per lesion than measuring its diameter. Additionally, as previously discussed, NETs tend not to shrink but rather stabilize in response to therapy and there is limited previously published data on volume measurements of liver metastases in NET patients. Notably, one earlier study on tumor volume assessment utilized a semi-automated method using a prototype software on patients undergoing ^177^Lu- and ^90^Y-DOTATOC combination therapy for NET liver metastases (38).

Therapy monitoring with CECT in the late arterial phase might also be questioned. While the sensitivity for detection of PNET liver metastases is higher in the arterial phase, both the inter- and intra-observer reproducibility of size measurement is likely to be higher when the lesions are assessed in the portal venous phase compared to the arterial phase (39). Although MRI is superior to CT for evaluation of liver lesions of NETs, both regarding accuracy and intra/inter-observer reproducibility, the generally much better availability of CT than of MRI, makes CT the primary imaging modality for NET therapy monitoring (3, 9–11, 39).

In conclusion, the arterial tumor attenuation decreased from baseline to follow-up, especially from mid-treatment to follow-up. There was an increase in tumor contrast-enhancement from baseline to mid-treatment, which correlated with PFS and with time to best response, indicating that these measurements can be useful in monitoring of PRRT in PNET patients, as a complement to tumor size-based evaluation according to the RECIST 1.1 criteria. There was a limited number of patients available for inclusion in our study and therefore, because of limited statistical power, a risk for underestimation of the fold changes of contrast-enhancement during PRRT. Thus, our findings need to be further evaluated, prospectively and in a larger patient cohort. Assessment in patients with other NET types and for other treatment regimens than PRRT could also be of interest.

## Data Availability Statement

The datasets generated for this study are available on request to the corresponding author.

## Ethics Statement

The study was approved by the Local Ethics Committee for Human Ethics in Uppsala, Sweden (reference number: 2010/177) and all patients provided written informed consent.

## Author Contributions

AS and OP: conception or design and manuscript writing. KF-B and AS: provision of patients. OP, KF-B, and AS: collection and/or assembly of data. OP, KF-B, JC, and AS: data analysis and interpretation and final approval of the manuscript.

### Conflict of Interest

AS has received lecture honoraria from Ipsen and honoraria for external imaging expert work from Advanced Accelerator Applications (AAA). JC has received lecture honoraria from Novartis and educational honoraria from NET Connect (funded by IPSEN). The remaining authors declare that the research was conducted in the absence of any commercial or financial relationships that could be construed as a potential conflict of interest.
